# Supplementary material for: Active vaccine safety surveillance: Experience from a prospective cohort event monitoring study of COVID-19 vaccines in Kenya
Source: PLOS Glob Public Health. 2025 Nov 17;5(11):e0005080. doi: 10.1371/journal.pgph.0005080 (PMC12622800; doi:10.1371/journal.pgph.0005080)
Supplement: S1 Table — Cells with a sample size of ≤10,000 are shaded. The calculation of the 95% CI if no event is observed is based on the methods proposed by Eypasch et al., 1995. (DOCX) [file pgph.0005080.s001.docx]

**S1 Table.** Sample sizes needed to rule out various levels of an increase in the risk of adverse events of special interest (AESI) if no event is observed within a 42-day risk window. Cells with a sample size of ≤10,000 are shaded. The calculation of the 95% CI if no event is observed is based on the methods proposed by Eypasch et al., 1995.

| **AESI** | **Background rate per 100,000 person years** | **Relative risk** | **Theoretical rate per 100,000 person years assuming increased risk (theoretical 95% CI)** | **Sample size** | **95% CI per 100,000 person years if no event is observed** |
| --- | --- | --- | --- | --- | --- |
| ARDS | 39 | 1.5 | 59 (45, 76) | 59,370 | 0, 44 |
|  |  | 2 | 78 (62, 97) | 42,983 | 0, 61 |
|  |  | 3 | 117 (97, 140) | 27,225 | 0, 96 |
|  |  | 4 | 156 (132, 182) | 19,829 | 0, 131 |
|  |  | 5 | 195 (169, 224) | 15,557 | 0, 168 |
|  | 90 | 1.5 | 135 (113, 160) | 23,239 | 0, 112 |
|  |  | 2 | 180 (155, 208) | 16,967 | 0, 154 |
|  |  | 3 | 270 (239, 304) | 10,966 | 0, 238 |
|  |  | 4 | 360 (324, 399) | 8,077 | 0, 323 |
|  |  | 5 | 450 (409, 494) | 6,384 | 0, 408 |
|  | 150 | 1.5 | 225 (197, 256) | 13,332 | 0, 196 |
|  |  | 2 | 300 (267, 336) | 9,801 | 0, 266 |
|  |  | 3 | 450 (409, 494) | 6,384 | 0, 408 |
|  |  | 4 | 600 (553, 650) | 4,724 | 0, 552 |
|  |  | 5 | 750 (697, 806) | 3,744 | 0, 696 |
|  | 193 | 1.5 | 290 (258, 325) | 10,161 | 0, 257 |
|  |  | 2 | 386 (348, 426) | 7,504 | 0, 347 |
|  |  | 3 | 579 (533, 628) | 4,903 | 0, 532 |
|  |  | 4 | 772 (718, 828) | 3,634 | 0, 717 |
|  |  | 5 | 965 (905, 1028) | 2,884 | 0, 904 |
| Thrombosis | 80 | 1.5 | 120 (99, 143) | 26,471 | 0, 98 |
|  |  | 2 | 160 (136, 187) | 19,288 | 0, 135 |
|  |  | 3 | 240 (211, 272) | 12,439 | 0, 210 |
|  |  | 4 | 320 (286, 357) | 9,151 | 0, 285 |
|  |  | 5 | 400 (362, 441) | 7,227 | 0, 361 |
| Acute aseptic arthritis | 100 | 1.5 | 150 (127, 176) | 20,699 | 0, 126 |
|  |  | 2 | 200 (173, 230) | 15,137 | 0, 172 |
|  |  | 3 | 300 (267, 336) | 9,801 | 0, 266 |
|  |  | 4 | 400 (362, 441) | 7,227 | 0, 361 |
|  |  | 5 | 500 (457, 546) | 5,716 | 0, 456 |
|  | 500 | 1.5 | 750 (697, 806) | 3,744 | 0, 696 |
|  |  | 2 | 1000 (939, 1064) | 2,780 | 0, 938 |
|  |  | 3 | 1500 (1425, 1578) | 1,831 | 0, 1,424 |
|  |  | 4 | 2000 (1913, 2090) | 1,363 | 0, 1,912 |
|  |  | 5 | 2500 (2403, 2600) | 1,085 | 0, 2,402 |
|  | 1000 | 1.5 | 1500 (1425, 1578) | 1,831 | 0, 1,424 |
|  |  | 2 | 2000 (1913, 2090) | 1,363 | 0, 1,912 |
|  |  | 3 | 3000 (2894, 3109) | 901 | 0, 2,893 |
|  |  | 4 | 4000 (3877, 4126) | 673 | 0, 3,876 |
|  |  | 5 | 1500 (1425, 1578) | 536 | 0, 4,861 |
|  | 1600 | 1.5 | 2400 (2305, 2498) | 1,132 | 2,304 |
|  |  | 2 | 3200 (3090, 3313) | 844 | 3,089 |
|  |  | 3 | 4800 (4665, 4938) | 559 | 4,664 |
|  |  | 4 | 6400 (6244, 6559) | 418 | 6,243 |
|  |  | 5 | 8000 (7826, 8177) | 333 | 7,825 |
