# Supplementary material for: Active vaccine safety surveillance: Experience from a prospective cohort event monitoring study of COVID-19 vaccines in Kenya
Source: PLOS Glob Public Health. 2025 Nov 17;5(11):e0005080. doi: 10.1371/journal.pgph.0005080 (PMC12622800; doi:10.1371/journal.pgph.0005080)
Supplement: S2 Table — Margins of error are based on the width of the exact binomial 95% confidence intervals for a proportion. (DOCX) [file pgph.0005080.s002.docx]

**S2 Table.** Margins of error for different levels of reactogenicity prevalence for a sample size of 1,000. Margins of error are based on the width of the exact binomial 95% confidence intervals for a proportion.

| **Prevalence estimate** | **Margin of error** |
| --- | --- |
| 1% | ±0.6% |
| 2% | ±0.8% |
| 5% | ±1.3% |
| 10% | ±1.9% |
| 25% | ±2.7% |
| 50% | ±3.1% |
