# Supplementary material for: Active vaccine safety surveillance: Experience from a prospective cohort event monitoring study of COVID-19 vaccines in Kenya
Source: PLOS Glob Public Health. 2025 Nov 17;5(11):e0005080. doi: 10.1371/journal.pgph.0005080 (PMC12622800; doi:10.1371/journal.pgph.0005080)
Supplement: S3 Table — (DOCX) [file pgph.0005080.s003.docx]

**S3 Table.** Distribution of vaccines received by brand and dose among enrolled participants at baseline (this excludes one re-enrolled participant with follow up over two doses).

| **Brand** | **Dose** | **Reactogenicity subset**  **N=1000**  **n (%)** | **Non-reactogenicity subset**  **N=1439**  **n (%)** | **Overall N=2439**  **n (%)** |
| --- | --- | --- | --- | --- |
| Pfizer | 1^st^ | 208 (20.8) | 267 (18.5) | 475 (19.5) |
|  | 2^nd^ | 108 (10.8) | 155 (10.8) | 263 (10.8) |
|  | 3^rd^ | 64 (6.4) | 75 (5.2) | 139 (5.7) |
|  | 4^th^ | 1 (0.1) | 2 (0.1) | 3 (0.1) |
| Johnson & Johnson | 1^st^ | 366 (36.6) | 445 (30.9) | 811 (33.3) |
|  | 2^nd^ | 87 (8.7) | 143 (9.9) | 230 (9.4) |
|  | 3^rd^ | 56 (5.6) | 64 (4.4) | 120 (4.9) |
|  | 4^th^ | 4 (0.4) | 2 (0.1) | 6 (0.2) |
| Moderna | 1^st^ | 28 (2.8) | 117 (8.1) | 145 (5.9) |
|  | 2^nd^ | 45 (4.5) | 98 (6.8) | 143 (5.9) |
|  | 3^rd^ | 28 (2.8) | 66 (4.6) | 94 (3.9) |
|  | 4^th^ | 5 (0.5) | 5 (0.3) | 10 (0.4) |
