# Supplementary material for: Active vaccine safety surveillance: Experience from a prospective cohort event monitoring study of COVID-19 vaccines in Kenya
Source: PLOS Glob Public Health. 2025 Nov 17;5(11):e0005080. doi: 10.1371/journal.pgph.0005080 (PMC12622800; doi:10.1371/journal.pgph.0005080)
Supplement: S4 Table — (DOCX) [file pgph.0005080.s004.docx]

**S4 Table**. Summary of follow up periods within the cohort.

| **Study week number** | **Number of participants for whom this was the terminal observation period** | **Percent** |
| --- | --- | --- |
| 1 | 15 | 0.6 |
| 2 | 12 | 0.5 |
| 3 | 10 | 0.4 |
| 4 | 15 | 0.6 |
| 5 | 8 | 0.3 |
| 6 | 11 | 0.5 |
| 7 | 7 | 0.3 |
| 8 | 13 | 0.5 |
| 9 | 25 | 1.1 |
| 10 | 20 | 0.8 |
| 11 | 23 | 1.0 |
| 12 | 12 | 0.5 |
| 13 | 2,183 | 92.7 |
| **Total** | 2,354 | 100 |
