# Supplementary material for: Active vaccine safety surveillance: Experience from a prospective cohort event monitoring study of COVID-19 vaccines in Kenya
Source: PLOS Glob Public Health. 2025 Nov 17;5(11):e0005080. doi: 10.1371/journal.pgph.0005080 (PMC12622800; doi:10.1371/journal.pgph.0005080)
Supplement: S5 Table — P-values compare the frequency of the event post-vaccination to baseline. Day 0 is the day of vaccination. (DOCX) [file pgph.0005080.s005.docx]

**S5 Table.** Frequency of systemic reactogenicity at baseline (within three days prior to vaccination) and daily during the first week post-vaccination. P-values compare the frequency of the event post-vaccination to baseline. Day 0 is the day of vaccination.

|  | **Chills** | | **Fatigue** | | **Fever** | | **Headache** | |
| --- | --- | --- | --- | --- | --- | --- | --- | --- |
|  | **% (95% CI)** | **p-value** | **% (95% CI)** | **p-value** | **% (95% CI)** | **p-value** | **% (95% CI)** | **p-value** |
| **Day -3 to -1 (N= 956)** | 8.6 (6.8-10.3) | Ref | 9.4 (7.6-11.3) | Ref | 5.7 (4.3-7.2) | Ref | 22.8 (20.1-25.5) | Ref |
| **Day 0 (N= 893)** | 15.3 (13.0-17.7) | **<0.001** | 24.1 (21.3-26.9) | **<0.001** | 13.9 (11.6-16.2) | **<0.001** | 21.3 (18.6-24.0) | 0.425 |
| **Day 1 (N= 884)** | 13.1 (10.9-15.3) | **<0.001** | 25.5 (22.6-28.3) | **<0.001** | 12.0 (9.8-14.1) | **<0.001** | 19.5 (16.8-22.1) | 0.103 |
| **Day 2 (N= 885)** | 6.9 (5.2-8.6) | 0.228 | 14.5 (12.1-16.8) | **0.001** | 5.4 (3.9-6.9) | 0.834 | 12.0 (9.8-14.1) | **<0.001** |
| **Day 3 (N= 881)** | 4.8 (3.4-6.2) | **0.005** | 10.6 (8.5-12.6) | 0.292 | 2.7 (1.6-3.8) | **0.001** | 9.1 (7.2-11.0) | **<0.001** |
| **Day 4 (N= 876)** | 3.1 (1.9-4.2) | **<0.001** | 7.1 (5.4-8.8) | 0.090 | 3.7 (2.4-4.9) | **0.034** | 7.6 (5.9-9.4) | **<0.001** |
| **Day 5 (N= 874)** | 3.1 (1.9-4.2) | **<0.001** | 6.2 (4.6-7.8) | **0.018** | 2.3 (1.3-3.3) | **<0.001** | 5.3 (3.8-6.7) | **<0.001** |
| **Day 6 (N= 890)** | 2.6 (1.5-3.6) | **<0.001** | 5.4 (3.9-6.9) | **0.002** | 1.9 (1.0-2.8) | **<0.001** | 6.5 (4.9-8.1) | **<0.001** |

|  | **Joint pain** | | **Malaise** | | **Muscle aches** | | **Nausea** | |
| --- | --- | --- | --- | --- | --- | --- | --- | --- |
|  | **% (95% CI)** | **p-value** | **% (95% CI)** | **p-value** | **% (95% CI)** | **p-value** | **% (95% CI)** | **p-value** |
| **Day -3 to -1 (N= 956)** | 11.3 (9.3-13.3) | Ref | 10.2 (8.3-12.2) | Ref | 5.9 (4.4-7.3) | Ref | 4.4 (3.1-5.7) | Ref |
| **Day 0 (N= 893)** | 16.7 (14.2-19.1) | **0.001** | 20.5 (17.8-23.1) | **<0.001** | 11.5 (9.4-13.6) | **<0.001** | 7.6 (5.9-9.4) | **0.006** |
| **Day 1 (N= 884)** | 16.1 (13.6-18.5) | **0.001** | 19.7 (17.1-22.3) | **<0.001** | 11.5 (9.4-13.6) | **<0.001** | 7.9 (6.1-9.7) | **0.001** |
| **Day 2 (N= 885)** | 9.4 (7.5-11.3) | 0.129 | 9.8 (7.9-11.8) | 0.932 | 6.4 (4.8-8.1) | 0.764 | 5.8 (4.2-7.3) | 0.254 |
| **Day 3 (N= 881)** | 5.8 (4.2-7.3) | **<0.001** | 7.0 (5.3-8.7) | **0.027** | 4.4 (3.1-5.8) | 0.314 | 3.4 (2.2-4.6) | 0.313 |
| **Day 4 (N= 876)** | 4.5 (3.1-5.8) | **<0.001** | 4.5 (3.1-5.8) | **<0.001** | 3.8 (2.5-5.0) | 0.053 | 2.2 (1.2-3.1) | **0.013** |
| **Day 5 (N= 874)** | 3.2 (2.0-4.4) | **<0.001** | 3.3 (2.1-4.5) | **<0.001** | 2.2 (1.2-3.1) | **<0.001** | 2.7 (1.7-3.8) | 0.058 |
| **Day 6 (N= 890)** | 2.9 (1.8-4.0) | **<0.001** | 3.8 (2.6-5.1) | **<0.001** | 2.2 (1.3-3.2) | **<0.001** | 1.8 (0.9-2.7) | **0.001** |
