# Supplementary material for: Active vaccine safety surveillance: Experience from a prospective cohort event monitoring study of COVID-19 vaccines in Kenya
Source: PLOS Glob Public Health. 2025 Nov 17;5(11):e0005080. doi: 10.1371/journal.pgph.0005080 (PMC12622800; doi:10.1371/journal.pgph.0005080)
Supplement: S6 Table — (DOCX) [file pgph.0005080.s006.docx]

**S6 Table.** Summary of systemic reactogenicity events reported within the first week of COVID-19 vaccination**.**

| **Systemic reactogenicity event** | **N=956**  **n with event (%)** | **Median duration of events in days (IQR)** |
| --- | --- | --- |
| Fatigue | 422(44.1) | 2(1-3) |
| Headache | 370(38.7) | 2(1-2) |
| Malaise | 346(36.2) | 1(1-2) |
| Joint Pain | 308(32.2) | 1(1-2) |
| Muscle Aches | 247(25.8) | 1(1-2) |
| Chills | 246(25.7) | 1(1-2) |
| Fever | 233(24.4) | 1(1-2) |
| Nausea | 171(17.9) | 1(1-2) |
| Any systemic reactogenicity event ^a^ | 595(62.2) | 2(1-3) |
| None ^b^ | 361(37.8) | .. |

Abbreviation: IQR, Interquartile range.  ^a^ Any systemic reactogenicity event denotes participants who reported at least one systemic reactogenicity event. ^b^ None denotes participants who did not report any systemic reactogenicity event.
