# Supplementary material for: Active vaccine safety surveillance: Experience from a prospective cohort event monitoring study of COVID-19 vaccines in Kenya
Source: PLOS Glob Public Health. 2025 Nov 17;5(11):e0005080. doi: 10.1371/journal.pgph.0005080 (PMC12622800; doi:10.1371/journal.pgph.0005080)
Supplement: S7 Table — (DOCX) [file pgph.0005080.s007.docx]

**S7 Table.** Stratification of systemic reactogenicity events by severity.

| **Systemic reactogenicity event** | **None^a^**  **n (%)** | **Mild^b^**  **n (%)** | **Moderate^c^**  **n (%)** | **Severe^d^**  **n (%)** |
| --- | --- | --- | --- | --- |
| Any Event^e^ | 369 (38.6) | 295 (30.9) | 217 (22.7) | 75 (7.9) |
| Chills | 710 (74.3) | 143 (15.0) | 72 (7.53) | 31 (3.2) |
| Fatigue | 534 (55.9) | 243 (25.4) | 135 (14.1) | 44 (4.6) |
| Headache | 586 (61.3) | 210 (22.0) | 116 (12.1) | 44 (4.6) |
| Joint pain | 648 (67.8) | 196 (20.5) | 80 (8.4) | 32 (3.4) |
| Malaise | 610 (63.8) | 195 (20.4) | 103 (10.8) | 48 (5.0) |
| Muscle aches | 709 (74.2) | 156 (16.3) | 64 (6.7) | 27 (2.8) |
| Nausea | 785 (82.1) | 114 (11.9) | 35 (3.7) | 22 (2.3) |

^a^ None denotes participants who did not report any systemic reactogenicity event^. e^Any event includes all the systemic reactogenicity events except fever. Participants who reported fever only (8 participants) were considered to have reported no event in this analysis. Fever was not ranked by severity since its severity was not solicited during the follow up period. The severity of the systemic reactogenicity events was determined based on their ability to interfere with the normal daily activities of the participants. ^b^Mild events did not interfere with the normal daily activities of participants. ^c^Moderate events somewhat interfered with the normal daily activities of participants. ^d^Severe events were considerable and prevented the normal daily activities of participants.
