# Supplementary material for: Active vaccine safety surveillance: Experience from a prospective cohort event monitoring study of COVID-19 vaccines in Kenya
Source: PLOS Glob Public Health. 2025 Nov 17;5(11):e0005080. doi: 10.1371/journal.pgph.0005080 (PMC12622800; doi:10.1371/journal.pgph.0005080)
Supplement: S8 Table — (DOCX) [file pgph.0005080.s008.docx]

**S8 Table.** Analysis of factors associated with fever

| **Baseline sociodemographic characteristic** | | **Fever** | | **Univariate analysis** | | | **Multivariate analysis^a^** | | |
| --- | --- | --- | --- | --- | --- | --- | --- | --- | --- |
|  | | **n^d^** | **%** | **Odds ratio** | **95% CI** | **p-value^b^** | **Odds ratio** | **95% CI** | **p-value^b^** |
| Age | 17-39yrs. | 158/672 | 23.5 | 1 | 1 | .. | 1 | 1 | .. |
|  | 40-59yrs. | 55/216 | 25.5 | 1.11 | (0.78-1.58) | 0.559 | 0.95 | (0.64-1.40) | 0.792 |
|  | 60+yrs. | 20/68 | 29.4 | 1.36 | (0.78-2.35) | 0.28 | 1.12 | (0.61-2.04) | 0.717 |
| Sex | Male | 58/223 | 26.0 | 1 | 1 | .. | 1 | 1 | .. |
|  | Female, not pregnant | 134/523 | 25.6 | 0.98 | (0.69-1.40) | 0.912 | 1.09 | (0.75-1.59) | 0.648 |
|  | Female, pregnant | 41/210 | 19.5 | 0.69 | (0.44-1.09) | 0.109 | 1.22 | (0.68-2.16) | 0.506 |
| Dose | 1 dose | 126/573 | 22.0 | 1 | 1 | .. | 1 | 1 | .. |
|  | 2 doses, no product mixing^c^ | 27/101 | 27.0 | 1.29 | (0.80-2.10) | 0.295 | 1.36 | (0.83-2.24) | 0.223 |
|  | 2 doses, product mixing^c^ | 32/127 | 25.0 | 1.20 | (0.76-1.87) | 0.434 | 1.24 | (0.76-2.03) | 0.398 |
|  | 3 doses, no product mixing^c^ | 7/30 | 23.0 | 1.08 | (0.45-2.57) | 0.863 | 1.67 | (0.67-4.20) | 0.275 |
|  | 3 doses, product mixing^c^ | 38/116 | 33.0 | 1.73 | (1.12-2.67) | **0.014** | 1.64 | (1.03-2.59) | 0.036 |
|  | 4 doses, product mixing^c^ | 3/9 | 33.0 | 1.77 | (0.44-7.19) | 0.422 | 1.31 | (0.30-5.74) | 0.722 |
| Brand | Pfizer | 62/364 | 17.0 | 1 | 1 | .. | 1 | 1 | .. |
|  | Johnson | 134/492 | 27.2 | 1.82 | (1.30-2.56) | **<0.001** | 2.14 | (1.36-3.36) | **0.001** |
|  | Moderna | 37/100 | 37.0 | 2.86 | (1.75-4.67) | **<0.001** | 2.75 | (1.62-4.68) | **<0.001** |
| Comorbidity | No | 166/691 | 24.0 | 1 | 1 | .. | 1 | 1 | .. |
|  | Yes | 67/265 | 25.3 | 1.07 | (0.77-1.49) | 0.685 | 0.94 | (0.65-1.36) | 0.735 |

Abbreviations: CI, confidence interval; yrs, years. Logistic regression model was used for both univariate and multivariate analysis. ^a^ Multivariate analysis adjusted for all variables in the table. ^b^ P<0.05 was considered statistically significant. ^c^ Product mixing refers to participants who received more than one vaccine brand. The total number of participants was 956. ^d^ n denotes the number of participants who reported fever.
