# Supplementary material for: Active vaccine safety surveillance: Experience from a prospective cohort event monitoring study of COVID-19 vaccines in Kenya
Source: PLOS Glob Public Health. 2025 Nov 17;5(11):e0005080. doi: 10.1371/journal.pgph.0005080 (PMC12622800; doi:10.1371/journal.pgph.0005080)
Supplement: S9 Table — (DOCX) [file pgph.0005080.s009.docx]

**S9 Table.** Analysis of factors associated with nausea

| **Baseline sociodemographic characteristic** | | **Nausea** |  | **Univariate analysis** | | | **Multivariate analysis^a^** | | |
| --- | --- | --- | --- | --- | --- | --- | --- | --- | --- |
|  |  | **n^d^** | **%** | **Odds ratio** | **95% CI** | **p-value^b^** | **Odds ratio** | **95% CI** | **p-value^b^** |
| Age | 17-39yrs. | 127/672 | 18.9 | 1 | 1 | .. | 1 | 1 | .. |
|  | 40-59yrs. | 39/216 | 18.1 | 0.95 | (0.64-1.41) | 0.782 | 0.88 | (0.56-1.39) | 0.588 |
|  | 60+yrs. | 5/68 | 7.3 | 0.34 | (0.13-0.86) | **0.023** | 0.38 | (0.14-1.02) | 0.054 |
| Sex | Male | 17/223 | 7.6 | 1 | 1 | .. | 1 | 1 | .. |
|  | Female, not pregnant | 103/523 | 19.7 | 2.97 | (1.73-5.10) | **<0.001** | 2.86 | (1.64-4.98) | **<0.001** |
|  | Female, pregnant | 51/210 | 24.3 | 3.89 | (2.16-6.99) | **<0.001** | 5.62 | (2.75-11.49) | **<0.001** |
| Dose | 1 dose | 101/573 | 17.6 | 1 | 1 | .. | 1 | 1 | .. |
|  | 2 doses, no product mixing^c^ | 17/101 | 16.8 | 0.95 | (0.54-1.66) | 0.846 | 0.92 | (0.51-1.65) | 0.777 |
|  | 2 doses, product mixing^c^ | 21/127 | 16.5 | 0.93 | (0.55-1.55) | 0.769 | 0.87 | (0.49-1.54) | 0.636 |
|  | 3 doses, no product mixing^c^ | 11/30 | 36.7 | 2.71 | (1.25-5.86) | 0.012 | 3.13 | (1.34-7.29) | **0.008** |
|  | 3 doses, product mixing^c^ | 21/116 | 18.1 | 1.03 | (0.62-1.74) | 0.902 | 1.07 | (0.62-1.87) | 0.800 |
|  | 4 doses, product mixing^c^ | 0/9 | 0 | 1 | 1 | .. | 1 | 1 | .. |
| Brand | Pfizer | 66/364 | 18.1 | 1 | 1 | .. | 1 | 1 | .. |
|  | Johnson & Johnson | 81/492 | 16.5 | 0.89 | (0.62-1.27) | 0.522 | 1.73 | (1.03-2.9) | **0.037** |
|  | Moderna | 24/100 | 24 | 1.43 | (0.84-2.42) | 0.190 | 2.31 | (1.28-4.2) | **0.006** |
| Comorbidity | No | 116/691 | 16.8 | 1 | 1 | .. | 1 | 1 | .. |
|  | Yes | 55/265 | 20.8 | 1.30 | (0.91-1.86) | 0.153 | 1.68 | (1.10-2.57) | **0.017** |

Abbreviations: CI, confidence interval; yrs, years. Logistic regression model was used for both univariate and multivariate analysis. ^a^ Multivariate analysis adjusted for all variables in the table. ^b^ P<0.05 was considered statistically significant. ^c^ Product mixing refers to participants who received more than one vaccine brand. The total number of participants was 956. ^d^ n denotes the number of participants who reported nausea.
