# Supplementary material for: Active vaccine safety surveillance: Experience from a prospective cohort event monitoring study of COVID-19 vaccines in Kenya
Source: PLOS Glob Public Health. 2025 Nov 17;5(11):e0005080. doi: 10.1371/journal.pgph.0005080 (PMC12622800; doi:10.1371/journal.pgph.0005080)
Supplement: S10 Table — (DOCX) [file pgph.0005080.s010.docx]

**S10 Table.** Analysis of factors associated with malaise.

| **Baseline sociodemographic characteristic** | | **Malaise** | | **Univariate analysis** | | | **Multivariate analysis^a^** | | |
| --- | --- | --- | --- | --- | --- | --- | --- | --- | --- |
|  | | **n^d^** | **%** | **Odds ratio** | **95% CI** | **p-value^b^** | **Odds ratio** | **95% CI** | **p-value^b^** |
| Age | 17-39yrs. | 250/672 | 37.2 | 1 | 1 | .. | 1 | 1 | .. |
|  | 40-59yrs. | 79/216 | 36.6 | 0.97 | (0.71-1.34) | 0.868 | 0.76 | (0.53-1.09) | 0.134 |
|  | 60+yrs. | 17/68 | 25.0 | 0.56 | (0.32-1.00) | **0.048** | 0.47 | (0.25-0.86) | **0.015** |
| Sex | Male | 69/223 | 30.9 | 1 | 1 | .. | 1 | 1 | .. |
|  | Female, not pregnant | 210/523 | 40.2 | 1.50 | (1.07-2.09) | **0.018** | 1.57 | (1.10-2.23) | 0.012 |
|  | Female, pregnant | 67/210 | 31.9 | 1.05 | (0.70-1.57) | 0.829 | 1.55 | (0.92-2.59) | 0.098 |
| Dose | 1 dose | 205/573 | 35.8 | 1 | 1 | .. | 1 | 1 | .. |
|  | 2 doses, no product mixing^c^ | 34/101 | 33.7 | 0.91 | (0.58-1.42) | 0.682 | 0.92 | (0.58-1.46) | 0.721 |
|  | 2 doses, product mixing^c^ | 49/127 | 38.6 | 1.13 | (0.76-1.68) | 0.552 | 1.08 | (0.70-1.68) | 0.725 |
|  | 3 doses, no product mixing^c^ | 12/30 | 40.0 | 1.20 | (0.57-2.53) | 0.639 | 1.56 | (0.71-3.47) | 0.272 |
|  | 3 doses, product mixing^c^ | 44/116 | 37.9 | 1.10 | (0.73-1.66) | 0.660 | 1.03 | (0.66-1.59) | 0.911 |
|  | 4 doses, product mixing^c^ | 2/9 | 22.2 | 0.51 | (0.11-2.49) | 0.408 | 0.60 | (0.12-3.12) | 0.542 |
| Brand | Pfizer | 104/364 | 28.6 | 1 | 1 | .. | 1 | 1 | .. |
|  | Johnson & Johnson | 191/492 | 38.8 | 1.59 | (1.19-2.12) | **0.002** | 1.99 | (1.34-2.96) | **0.001** |
|  | Moderna | 51/100 | 51.0 | 2.60 | (1.65-4.09) | **<0.001** | 3.06 | (1.87-5.02) | **<0.001** |
| Comorbidity | No | 241/691 | 34.9 | 1 | 1 | .. | 1 | 1 | .. |
|  | Yes | 105/265 | 39.6 | 1.23 | (0.92-1.64) | 0.172 | 1.30 | (0.93-1.82) | 0.127 |

Abbreviations: CI, confidence interval; yrs, years. Logistic regression model was used for both univariate and multivariate analysis. ^a^ Multivariate analysis adjusted for all variables in the table. ^b^ P<0.05 was considered statistically significant. ^c^ Product mixing refers to participants who received more than one vaccine brand. The total number of participants was 956. ^d^ n denotes the number of participants who reported malaise.
