# Supplementary material for: Active vaccine safety surveillance: Experience from a prospective cohort event monitoring study of COVID-19 vaccines in Kenya
Source: PLOS Glob Public Health. 2025 Nov 17;5(11):e0005080. doi: 10.1371/journal.pgph.0005080 (PMC12622800; doi:10.1371/journal.pgph.0005080)
Supplement: S11 Table — (DOCX) [file pgph.0005080.s011.docx]

**S11 Table.** Analysis of factors associated with chills.

| **Baseline sociodemographic characteristic** | | **Chills** | | **Univariate analysis** | | | **Multivariate analysis^a^** | | |
| --- | --- | --- | --- | --- | --- | --- | --- | --- | --- |
|  | | **n^d^** | **%** | **Odds ratio** | **95% CI** | **p-value^b^** | **Odds ratio** | **95% CI** | **p-value^b^** |
| Age | 17-39yrs. | 177/672 | 26.3 | 1 | 1 | .. | 1 | 1 | .. |
|  | 40-59yrs. | 57/216 | 26.4 | 1.00 | (0.71-1.42) | 0.989 | 0.78 | (0.53-1.15) | 0.214 |
|  | 60+yrs. | 12/68 | 17.6 | 0.60 | (0.31-1.14) | 0.121 | 0.53 | (0.26-1.05) | 0.069 |
| Sex | Male | 46/223 | 20.6 | 1 | 1 | .. | 1 | 1 | .. |
|  | Female, not pregnant | 163/523 | 31.2 | 1.74 | (1.20-2.53) | **0.004** | 1.86 | (1.26-2.76) | **0.002** |
|  | Female, pregnant | 37/210 | 17.6 | 0.82 | (0.51-1.33) | 0.427 | 1.30 | (0.72-2.36) | 0.382 |
| Dose | 1 dose | 138/573 | 24.1 | 1 | 1 | **..** | 1 | 1 | **..** |
|  | 2 doses, no product mixing^c^ | 18/101 | 17.8 | 0.68 | (0.40-1.18) | 0.171 | 0.68 | (0.39-1.20) | 0.183 |
|  | 2 doses, product mixing^c^ | 35/127 | 27.6 | 1.20 | (0.78-1.85) | 0.412 | 1.11 | (0.68-1.83) | 0.668 |
|  | 3 doses, no product mixing^c^ | 13/30 | 43.3 | 2.41 | (1.14-5.09) | **0.021** | 3.48 | (1.54-7.88) | **0.003** |
|  | 3 doses, product mixing^c^ | 41/116 | 35.3 | 1.72 | (1.13-2.64) | **0.012** | 1.53 | (0.97-2.43) | 0.069 |
|  | 4 doses, product mixing^c^ | 1/9 | 11.1 | 0.39 | (0.05-3.18) | 0.382 | 0.37 | (0.04-3.19) | 0.365 |
| Brand | Pfizer | 63/364 | 17.3 | 1 | 1 | .. | 1 | 1 | .. |
|  | Johnson & Johnson | 139/492 | 28.3 | 1.88 | (1.35-2.63) | **<0.001** | 2.41 | (1.53-3.78) | **<0.001** |
|  | Moderna | 44/100 | 44.0 | 3.75 | (2.33-6.06) | **<0.001** | 4.11 | (2.42-6.98) | **<0.001** |
| Comorbidity | No | 175/691 | 25.3 | 1 | 1 | .. | 1 | 1 | .. |
|  | Yes | 71/265 | 26.8 | 1.08 | (0.78-1.49) | 0.642 | 1.04 | (0.72-1.52) | 0.82 |

Abbreviations: CI, confidence interval; yrs, years. Logistic regression model was used for both univariate and multivariate analysis. ^a^ Multivariate analysis adjusted for all variables in the table. ^b^ P<0.05 was considered statistically significant. ^c^ Product mixing refers to participants who received more than one vaccine brand. The total number of participants was 956. ^d^ n denotes the number of participants who reported chills.
