# Supplementary material for: Active vaccine safety surveillance: Experience from a prospective cohort event monitoring study of COVID-19 vaccines in Kenya
Source: PLOS Glob Public Health. 2025 Nov 17;5(11):e0005080. doi: 10.1371/journal.pgph.0005080 (PMC12622800; doi:10.1371/journal.pgph.0005080)
Supplement: S12 Table — (DOCX) [file pgph.0005080.s012.docx]

**S12 Table.** Analysis of factors associated with headache.

| **Baseline sociodemographic characteristic** | | **Headache** |  | **Univariate analysis** | | | **Multivariate analysis^a^** | | |
| --- | --- | --- | --- | --- | --- | --- | --- | --- | --- |
|  | | **n^d^** | **%** | **Odds ratio** | **95% CI** | **p-value^b^** | **Odds ratio** | **95% CI** | **p-value^b^** |
| Age | 17-39yrs. | 262/672 | 39.0 | 1 | 1 | .. | 1 | 1 | .. |
|  | 40-59yrs. | 88/216 | 40.7 | 1.08 | (0.79-1.47) | 0.647 | 0.87 | (0.61-1.24) | 0.443 |
|  | 60+yrs. | 20/68 | 29.4 | 0.65 | (0.39-1.12) | 0.123 | 0.52 | (0.29-0.94) | **0.030** |
| Sex | Male | 69/223 | 30.9 | 1 | 1 | .. | 1 | 1 | .. |
|  | Female, not pregnant | 230/523 | 44.0 | 1.75 | (1.26-2.44) | **0.001** | 1.79 | (1.26-2.54) | **0.001** |
|  | Female, pregnant | 71/210 | 33.8 | 1.14 | (0.76-1.71) | 0.524 | 1.53 | (0.92-2.55) | 0.099 |
| Dose | 1 dose | 212/573 | 37.0 | 1 | 1 | .. | 1 | 1 | .. |
|  | 2 doses, no product mixing^c^ | 34/101 | 33.7 | 0.86 | (0.55-1.35) | 0.521 | 0.82 | (0.52-1.30) | 0.405 |
|  | 2 doses, product mixing^c^ | 49/127 | 38.6 | 1.07 | (0.72-1.59) | 0.738 | 0.96 | (0.62-1.48) | 0.837 |
|  | 3 doses, no product mixing^c^ | 15/30 | 50.0 | 1.70 | (0.82-3.55) | 0.156 | 1.86 | (0.85-4.04) | 0.119 |
|  | 3 doses, product mixing^c^ | 56/116 | 48.3 | 1.59 | (1.06-2.38) | **0.024** | 1.48 | (0.97-2.27) | 0.072 |
|  | 4 doses, product mixing^c^ | 4/9 | 44.4 | 1.36 | (0.36-5.13) | 0.648 | 1.70 | (0.41-6.96) | 0.464 |
| Brand | Pfizer | 121/364 | 33.2 | 1 | 1 | .. | 1 | 1 | .. |
|  | Johnson & Johnson | 195/492 | 39.6 | 1.32 | (0.99-1.75) | 0.056 | 1.50 | (1.02-2.19) | **0.040** |
|  | Moderna | 54/100 | 54.0 | 2.36 | (1.50-3.70) | **<0.001** | 2.49 | (1.53-4.06) | **<0.001** |
| Comorbidity | No | 255/691 | 36.9 | 1 | 1 | .. | 1 | 1 | .. |
|  | Yes | 115/265 | 43.4 | 1.31 | (0.98-1.75) | 0.065 | 1.40 | (1.00-1.96) | **0.048** |

Abbreviations: CI, confidence interval; yrs, years. Logistic regression model was used for both univariate and multivariate analysis. ^a^ Multivariate analysis adjusted for all variables in the table. ^b^ P<0.05 was considered statistically significant. ^c^ Product mixing refers to participants who received more than one vaccine brand. The total number of participants was 956. ^d^ n denotes the number of participants who reported headache.
