# Supplementary material for: Active vaccine safety surveillance: Experience from a prospective cohort event monitoring study of COVID-19 vaccines in Kenya
Source: PLOS Glob Public Health. 2025 Nov 17;5(11):e0005080. doi: 10.1371/journal.pgph.0005080 (PMC12622800; doi:10.1371/journal.pgph.0005080)
Supplement: S13 Table — (DOCX) [file pgph.0005080.s013.docx]

**S13 Table.** Analysis of factors associated with joint pain.

| **Baseline sociodemographic characteristic** | | **Joint pain** |  | **Univariate analysis** | | | **Multivariate analysis^a^** | | |
| --- | --- | --- | --- | --- | --- | --- | --- | --- | --- |
|  | | **n^d^** | **%** | **Odds ratio** | **95% CI** | **p-value^b^** | **Odds ratio** | **95% CI** | **p-value^b^** |
| Age | 17-39yrs. | 221/672 | 32.9 | 1 | 1 | .. | 1 | 1 | .. |
|  | 40-59yrs. | 68/216 | 31.5 | 0.94 | (0.68-1.30) | 0.701 | 0.76 | (0.52-1.09) | 0.138 |
|  | 60+yrs. | 19/68 | 27.9 | 0.79 | (0.46-1.38) | 0.407 | 0.71 | (0.39-1.28) | 0.253 |
| Sex | Male | 66/223 | 29.6 | 1 | 1 | .. | 1 | 1 | .. |
|  | Female, not pregnant | 180/523 | 34.4 | 1.25 | (0.89-1.75) | 0.200 | 1.35 | (0.94-1.93) | 0.100 |
|  | Female, pregnant | 62/210 | 29.5 | 1.00 | (0.66-1.51) | 0.987 | 1.65 | (0.97-2.81) | 0.066 |
| Dose | 1 dose | 181/573 | 31.6 | 1 | 1 | .. | 1 | 1 | .. |
|  | 2 doses, no product mixing^c^ | 26/101 | 25.7 | 0.75 | (0.47-1.21) | 0.241 | 0.77 | (0.47-1.25) | 0.287 |
|  | 2 doses, product mixing^c^ | 46/127 | 36.2 | 1.23 | (0.82-1.84) | 0.313 | 1.17 | (0.74-1.84) | 0.497 |
|  | 3 doses, no product mixing^c^ | 9/30 | 30.0 | 0.93 | (0.42-2.07) | 0.855 | 1.35 | (0.58-3.17) | 0.489 |
|  | 3 doses, product mixing^c^ | 45/116 | 38.8 | 1.37 | (0.91-2.07) | 0.133 | 1.27 | (0.82-1.97) | 0.288 |
|  | 4 doses, product mixing^c^ | 1/9 | 11.1 | 0.27 | (0.03-2.18) | 0.220 | 0.23 | (0.03-1.98) | 0.181 |
| Brand | Pfizer | 87/364 | 23.9 | 1 | 1 | .. | 1 | 1 | .. |
|  | Johnson & Johnson | 170/492 | 34.5 | 1.68 | (1.24-2.28) | **0.001** | 2.21 | (1.46-3.36) | **<0.001** |
|  | Moderna | 51/100 | 51.0 | 3.31 | (2.09-5.25) | **<0.001** | 3.80 | (2.30-6.30) | **<0.001** |
| Comorbidity | No | 216/691 | 31.3 | 1 | 1 | .. | 1 | 1 | .. |
|  | Yes | 92/265 | 34.7 | 1.17 | (0.87-1.58) | 0.306 | 1.24 | (0.88-1.75) | 0.227 |

Abbreviations: CI, confidence interval; yrs, years. Logistic regression model was used for both univariate and multivariate analysis. ^a^ Multivariate analysis adjusted for all variables in the table. ^b^ P<0.05 was considered statistically significant. ^c^ Product mixing refers to participants who received more than one vaccine brand. The total number of participants was 956. ^d^ n denotes the number of participants who reported joint pain.
