# Supplementary material for: Active vaccine safety surveillance: Experience from a prospective cohort event monitoring study of COVID-19 vaccines in Kenya
Source: PLOS Glob Public Health. 2025 Nov 17;5(11):e0005080. doi: 10.1371/journal.pgph.0005080 (PMC12622800; doi:10.1371/journal.pgph.0005080)
Supplement: S14 Table — (DOCX) [file pgph.0005080.s014.docx]

**S14 Table.** Analysis of factors associated with muscle aches.

| **Baseline sociodemographic characteristic** | | **Muscle aches** |  | **Univariate analysis** | | | **Multivariate analysis^a^** | | |
| --- | --- | --- | --- | --- | --- | --- | --- | --- | --- |
|  | | **n^d^** | **%** | **Odds ratio** | **95% CI** | **p-value^b^** | **Odds ratio** | **95% CI** | **p-value^b^** |
| Age | 17-39yrs. | 170/672 | 25.3 | 1 | 1 | .. | 1 | 1 | .. |
|  | 40-59yrs. | 59/216 | 27.3 | 1.11 | (0.79-1.57) | 0.556 | 0.83 | (0.57-1.22) | 0.352 |
|  | 60+yrs. | 18/68 | 26.5 | 1.06 | (0.60-1.87) | 0.832 | 0.81 | (0.44-1.49) | 0.494 |
| Sex | Male | 60/223 | 26.9 | 1 | 1 | .. | 1 | 1 | .. |
|  | Female, not pregnant | 156/523 | 29.8 | 1.16 | (0.81-1.64) | 0.421 | 1.20 | (0.83-1.74) | 0.324 |
|  | Female, pregnant | 31/210 | 14.8 | 0.47 | (0.29-0.76) | **0.002** | 0.67 | (0.38-1.21) | 0.188 |
| Dose | 1 dose | 134/573 | 23.4 | 1 | 1 | .. | 1 | 1 | .. |
|  | 2 doses, no product mixing^c^ | 24/101 | 23.8 | 1.02 | (0.62-1.68) | 0.934 | 1.01 | (0.61-1.69) | 0.958 |
|  | 2 doses, product mixing^c^ | 40/127 | 31.5 | 1.51 | (0.99-2.30) | 0.057 | 1.44 | (0.89-2.32) | 0.134 |
|  | 3 doses, no product mixing^c^ | 9/30 | 30.0 | 1.40 | (0.63-3.14) | 0.408 | 1.95 | (0.82-4.65) | 0.132 |
|  | 3 doses, product mixing^c^ | 38/116 | 32.8 | 1.60 | (1.04-2.46) | **0.034** | 1.36 | (0.86-2.16) | 0.193 |
|  | 4 doses, product mixing^c^ | 2/9 | 22.2 | 0.94 | (0.19-4.56) | 0.935 | 0.66 | (0.13-3.46) | 0.622 |
| Brand | Pfizer | 62/364 | 17.0 | 1 | 1 | .. | 1 | 1 | .. |
|  | Johnson & Johnson | 143/492 | 29.1 | 2.00 | (1.43-2.79) | **<0.001** | 1.89 | (1.22-2.92) | **0.004** |
|  | Moderna | 42/100 | 42.0 | 3.53 | (2.18-5.71) | **<0.001** | 3.11 | (1.85-5.23) | **<0.001** |
| Comorbidity | No | 171/691 | 24.8 | 1 | 1 | .. | 1 | 1 | .. |
|  | Yes | 76/265 | 28.7 | 1.22 | (0.89-1.68) | 0.214 | 1.08 | (0.75-1.56) | 0.664 |

Abbreviations: CI, confidence interval; yrs, years. Logistic regression model was used for both univariate and multivariate analysis. ^a^ Multivariate analysis adjusted for all variables in the table. ^b^ P<0.05 was considered statistically significant. ^c^ Product mixing refers to participants who received more than one vaccine brand. The total number of participants was 956. ^d^ n denotes the number of participants who reported muscle aches.
