# Supplementary material for: Active vaccine safety surveillance: Experience from a prospective cohort event monitoring study of COVID-19 vaccines in Kenya
Source: PLOS Glob Public Health. 2025 Nov 17;5(11):e0005080. doi: 10.1371/journal.pgph.0005080 (PMC12622800; doi:10.1371/journal.pgph.0005080)
Supplement: S15 Table — (DOCX) [file pgph.0005080.s015.docx]

**S15 Table.** Analysis of factors associated with fatigue.

| **Baseline sociodemographic characteristic** | | **Fatigue** |  | **Univariate analysis** | | | **Multivariate analysis^a^** | | |
| --- | --- | --- | --- | --- | --- | --- | --- | --- | --- |
|  | | **n^d^** | **%** | **Odds ratio** | **95% CI** | **p-value^b^** | **Odds ratio** | **95% CI** | **p-value^b^** |
| Age | 17-39yrs. | 302/672 | 44.9 | 1 | 1 | .. | 1 | 1 | .. |
|  | 40-59yrs. | 97/216 | 44.9 | 1.00 | (0.73-1.36) | 0.993 | 0.83 | (0.59-1.18) | 0.307 |
|  | 60+yrs. | 23/68 | 33.8 | 0.63 | (0.37-1.06) | **0.080** | 0.52 | (0.29-0.93) | **0.027** |
| Sex | Male | 87/223 | 39.0 | 1 | 1 | .. | 1 | 1 | .. |
|  | Female, not pregnant | 246/523 | 47.0 | 1.39 | (1.01-1.91) | **0.044** | 1.55 | (1.11-2.18) | **0.011** |
|  | Female, pregnant | 89/210 | 42.4 | 1.15 | (0.78-1.69) | 0.476 | 2.12 | (1.28-3.51) | **0.003** |
| Dose | 1 dose | 242/573 | 42.2 | 1 | 1 | .. | 1 | 1 | .. |
|  | 2 doses, no product mixing^c^ | 42/101 | 41.6 | 0.97 | (0.63-1.50) | 0.903 | 1.04 | (0.67-1.62) | 0.867 |
|  | 2 doses, product mixing^c^ | 63/127 | 49.6 | 1.35 | (0.92-1.98) | 0.130 | 1.33 | (0.86-2.05) | 0.199 |
|  | 3 doses, no product mixing^c^ | 13/30 | 43.3 | 1.05 | (0.50-2.19) | 0.905 | 1.57 | (0.71-3.47) | 0.261 |
|  | 3 doses, product mixing^c^ | 58/116 | 50.0 | 1.37 | (0.92-2.04) | 0.125 | 1.31 | (0.85-2.02) | 0.216 |
|  | 4 doses, product mixing^c^ | 4/9 | 44.4 | 1.09 | (0.29-4.12) | 0.894 | 1.11 | (0.27-4.64) | 0.882 |
| Brand | Pfizer | 125/364 | 34.3 | 1 | 1 | .. | 1 | 1 | .. |
|  | Johnson & Johnson | 229/492 | 46.5 | 1.67 | (1.26-2.20) | **<0.001** | 2.53 | (1.71-3.74) | **<0.001** |
|  | Moderna | 68/100 | 68.0 | 4.06 | (2.53-6.52) | **<0.001** | 4.75 | (2.83-7.96) | **<0.001** |
| Comorbidity | No | 302/691 | 43.7 | 1 | 1 | .. | 1 | 1 | .. |
|  | Yes | 120/265 | 45.3 | 1.07 | (0.80-1.42) | 0.660 | 1.11 | (0.80-1.55) | 0.526 |

Abbreviations: CI, confidence interval; yrs, years. Logistic regression model was used for both univariate and multivariate analysis. ^a^ Multivariate analysis adjusted for all variables in the table. ^b^ P<0.05 was considered statistically significant. ^c^ Product mixing refers to participants who received more than one vaccine brand. The total number of participants was 956. ^d^ n denotes the number of participants who reported fatigue.
