# Supplementary material for: Active vaccine safety surveillance: Experience from a prospective cohort event monitoring study of COVID-19 vaccines in Kenya
Source: PLOS Glob Public Health. 2025 Nov 17;5(11):e0005080. doi: 10.1371/journal.pgph.0005080 (PMC12622800; doi:10.1371/journal.pgph.0005080)
Supplement: S16 Table — (DOCX) [file pgph.0005080.s016.docx]

**S16 Table.** Summary of pregnancy related post-vaccination hospitalization events within the cohort.

|  | **Age in years** | **Reported event** | **Time of event onset in days relative to the date of vaccination** | **Vaccine name** | **Vaccine dose** |
| --- | --- | --- | --- | --- | --- |
| 1. | 38 | Non-reassuring fetal status | 11 | Pfizer | 1^st^ Vaccination |
| 2. | 33 | Incomplete abortion | 16 | Pfizer | 3^rd^ Vaccination |
| 3. | 28 | Incomplete abortion | 22 | Pfizer | 3^rd^ Vaccination |
| 4. | 24 | High blood pressure in pregnancy | 34 | Moderna | 2^nd^ Vaccination |
| 5. | 22 | Antepartum haemorrhage | 39 | Pfizer | 1^st^ Vaccination |
| 6. | 29 | Spontaneous abortion | 40 | Moderna | 2^nd^ Vaccination |
| 7. | 33 | Incomplete abortion | 45 | Pfizer | 2^nd^ Vaccination |
| 8. | 24 | High blood pressure in pregnancy | 55 | Moderna | 1^st^ Vaccination |
| 9. | 23 | Premature delivery | 57 | Pfizer | 1^st^ Vaccination |
| 10. | 27 | Threatened abortion | 66 | Pfizer | 2^nd^ Vaccination |
| 11. | 27 | Spontaneous abortion | 84 | Pfizer | 1^st^ Vaccination |
| 12. | 19 | Antepartum haemorrhage | 84 | Pfizer | 1^st^ Vaccination |
| 13. | 21 | Complicated pregnancy | 84 | Pfizer | 1^st^ Vaccination |
| 14. | 26 | Preterm premature rupture of membranes (PPROM) at 33 weeks of pregnancy | 88 | Moderna | 1^st^ Vaccination |
