# Supplementary material for: Active vaccine safety surveillance: Experience from a prospective cohort event monitoring study of COVID-19 vaccines in Kenya
Source: PLOS Glob Public Health. 2025 Nov 17;5(11):e0005080. doi: 10.1371/journal.pgph.0005080 (PMC12622800; doi:10.1371/journal.pgph.0005080)
Supplement: S17 Table — (DOCX) [file pgph.0005080.s017.docx]

**S17 Table.** Summary of elective surgeries reported as post-vaccination hospitalization events within the cohort.

|  | **Age** | **Reported event** | **Time of event onset in days relative to the date of vaccination** | **Vaccine name** | **Vaccine dose** |
| --- | --- | --- | --- | --- | --- |
| 1. | 68 | Inguinal hernia | 34 | Moderna | 2^nd^ Vaccination |
| 2. | 61 | Lipoma (Armpit region) | 47 | Johnson & Johnson | 2^nd^ Vaccination |
| 3. | 60 | Intestinal obstruction | 89 | Johnson & Johnson | 2^nd^ Vaccination |
