# Supplementary material for: Active vaccine safety surveillance: Experience from a prospective cohort event monitoring study of COVID-19 vaccines in Kenya
Source: PLOS Glob Public Health. 2025 Nov 17;5(11):e0005080. doi: 10.1371/journal.pgph.0005080 (PMC12622800; doi:10.1371/journal.pgph.0005080)
Supplement: S18 Table — (DOCX) [file pgph.0005080.s018.docx]

**S18 Table.** Summary of other medical conditions reported as post-vaccination hospitalization events within the cohort.

|  | **Age in years** | **Reported event(s)** | **Time of event onset in days relative to the date of vaccination** | **Vaccine name** | **Vaccine dose** |
| --- | --- | --- | --- | --- | --- |
| 1. | 32 | Dengue fever | 22 | Johnson & Johnson | 1^st^ Vaccination |
| 2. | 36 | High blood pressure | 27 | Pfizer | 2^nd^ Vaccination |
| 3. | 64 | Vomiting | 34 | Johnson & Johnson | 1^st^ Vaccination |
| 4. | 25 | Generalized malaise | 41 | Pfizer | 1^st^ Vaccination |
| 5. | 23 | Malaria | 44 | Johnson & Johnson | 1^st^ Vaccination |
| 6. | 26 | Dehydration | 49 | Johnson & Johnson | 3^rd^ Vaccination |
| 7. | 64 | Chronic kidney failure and cancer | 55 | Johnson & Johnson | 1^st^ Vaccination |
| 8. | 56 | Diarrhoea | 55 | Johnson & Johnson | 1^st^ Vaccination |
| 9. | 20 | Headache, malaise, and weakness | 89 | Pfizer | 1^st^ Vaccination |
| 10. | 20 | Headache, fever, and abdominal pain | 71 | Pfizer | 1^st^ Vaccination |
| 11. | 56 | Pneumonia | 77 | Moderna | 2^nd^ Vaccination |
| 12. | 47 | Cervical cancer | 80 | Johnson & Johnson | 1^st^ Vaccination |
| 13. | 21 | Malaria | 81 | Pfizer | 1^st^ Vaccination |
| 14. | 39 | Lung bulge | 105 | Pfizer | 1^st^ Vaccination |
