# Supplementary material for: Active vaccine safety surveillance: Experience from a prospective cohort event monitoring study of COVID-19 vaccines in Kenya
Source: PLOS Glob Public Health. 2025 Nov 17;5(11):e0005080. doi: 10.1371/journal.pgph.0005080 (PMC12622800; doi:10.1371/journal.pgph.0005080)
Supplement: S19 Table — (DOCX) [file pgph.0005080.s019.docx]

**S19 Table.** Non-hospitalization events reported by study participants

|  | **Age in years** | **Reported event (s)** | **Time of event onset in days relative to the date of vaccination** | **Vaccine name** | **Vaccine dose** |
| --- | --- | --- | --- | --- | --- |
| 1 | 25 | Joint pains, dizziness, and stoppage of menses | 0 | Johnson & Johnson | 1^st^ Booster |
| 2 | 50 | Headache and heart palpitations | 2 | Pfizer | 1^st^ Booster |
| 3 | 40 | Reduced breast milk production, and blurred vision | 3 | Johnson & Johnson | 1^st^ Vaccination |
| 4 | 25 | Left breast swelling | 4 | Johnson & Johnson | 1^st^ Vaccination |
| 5 | 34 | Itchiness of left foot followed by blister | 4 | Johnson & Johnson | 1st Vaccination |
| 6 | 53 | Itchiness and numbness of left lower limb | 52 | Johnson & Johnson | 1^st^ Vaccination |
