# Supplementary material for: Active vaccine safety surveillance: Experience from a prospective cohort event monitoring study of COVID-19 vaccines in Kenya
Source: PLOS Glob Public Health. 2025 Nov 17;5(11):e0005080. doi: 10.1371/journal.pgph.0005080 (PMC12622800; doi:10.1371/journal.pgph.0005080)
Supplement: S20 Table — (DOCX) [file pgph.0005080.s020.docx]

**S20 Table.** Analysis of SMS response rate among a subset of non-reactogenicity participants.

| **Week of follow-up** | **Participants targeted^a^**  **N=90** | **No. of participants with complete response^b^**  **n (%)** | **No. of participants with partial response^c^**  **n (%)** | **No. of participants who didn’t respond^d^**  **n (%)** | **No. of participants who responded incorrectly^e^**  **n (%)** |
| --- | --- | --- | --- | --- | --- |
| 1 | 16 | 8 (50.0) | 0 (0.0) | 0 (0.0) | 8 (50.0) |
| 2 | 20 | 8 (40.0) | 1 (5.0) | 0 (0.0) | 11 (55.0) |
| 3 | 20 | 10 (50.0) | 0 (0.0) | 0 (0.0) | 10 (50.0) |
| 4 | 36 | 10 (27.8) | 0 (0.0) | 4 (11.1) | 22 (61.1) |
| 5 | 73 | 25 (34.2) | 0 (0.0) | 10 (13.7) | 38 (52.1) |
| 6 | 94 | 26 (27.7) | 2 (2.1) | 10 (10.6) | 56 (59.6) |
| 7 | 91 | 29 (31.9) | 0 (0.0) | 10 (11.0) | 52 (57.1) |
| 8 | 89 | 32 (36.0) | 2 (2.2) | 13 (14.6) | 42 (47.2) |
| 9 | 87 | 29 (33.3) | 2 (2.3) | 31 (35.6) | 25 (28.7) |
| 10 | 83 | 32 (38.6) | 1 (1.2) | 43 (51.8) | 7 (8.4) |
| 11 | 69 | 27 (39.1) | 5 (7.2) | 33 (47.8) | 4 (5.8) |
| 12 | 68 | 23 (33.8) | 0 (0.0) | 41 (60.3) | 4 (5.9) |
| 13 | 68 | 21 (30.9) | 0 (0.0) | 41 (60.3) | 6 (8.8) |
| Total | 814 | 280 (34.4) | 13 (1.6) | 236 (29.0) | 285 (35.0) |

Abbreviations: SMS, Short Message Service; No., Number. A total of 90 non-reactogenicity participants were followed up using the SMS platform during their weekly follow-up (Week 1-13) to gather information on post-vaccination events. ^a^ Participants targeted denotes participants due for follow-up by SMS for that week. ^b^ No. of participants with complete response denotes participants who responded correctly to all the SMS sent to them. ^c^ No. of participants with partial response denotes participants who responded only to some of the SMS sent to them. ^d^ No. of participants who didn’t respond denotes participants who did not answer any of the SMS sent to them. ^e^ No. of participants who responded incorrectly denotes participants who provided invalid responses (wrong answers) to the SMS sent to them.
