# Supplementary material for: Active vaccine safety surveillance: Experience from a prospective cohort event monitoring study of COVID-19 vaccines in Kenya
Source: PLOS Glob Public Health. 2025 Nov 17;5(11):e0005080. doi: 10.1371/journal.pgph.0005080 (PMC12622800; doi:10.1371/journal.pgph.0005080)
Supplement: S1 Text — (PDF) [file pgph.0005080.s021.pdf]

CEM for COVID-19 vaccine safety

Baseline questionnaire: Participant registration, informed consent, contact, and covariates

Question	Response value/ coding or type
1. Unique identifier for each site	Unique Numeric/ text/ alphanumeric
2. Unique identifier for each participant	Unique Numeric/ text/ alphanumeric
3. Name of participant	
3.1 First name	Text
3.2 Middle name	Text
3.3 Surname	Text
4. Informed consent provided?	0 = No<br>1 = Yes
5. Is the participant part of the reactogenicity subset	0 = No<br>1 = Yes
6. Participant contact details	
6.1. Participant's phone number	Numeric
6.2. Name of next of kin	Text
6.3. Phone number of next of kin	Numeric
7. Participant covariates	
7.1. Date of birth	dd/mm/yyyy
7.2. Sex	0 = Male<br>1 = Female<br>2 = Other
7.3. Is the participant pregnant?	0 = No<br>1 = Yes
7.4. Is the participant breastfeeding?	0 = No<br>1 = Yes
7.5. Medical history, presence of diseases?	0 = No medical history<br>1 = Chronic respiratory disease or asthma<br>2 = Chronic heart disease<br>3 = Chronic liver disease<br>4 = Chronic renal disease<br>5 = Diabetes<br>6 = Immunocompromised/ immunosuppressed<br>7 = Obesity<br>8 = Allergy
7.6. Previous COVID-19 disease?	0 = No<br>1 = Yes, laboratory confirmed<br>2 = Probable but not laboratory-confirmed
7.7. History of reaction to any vaccination?	0 = No<br>1 = Yes<br>2 = Do not know

Question	Response value/ coding or type
7.7.1. **If yes**: Indicate which vaccine	1= BCG (Tuberculosis vaccine)<br>2= Polio vaccine<br>3=DPT (diphtheria, pertussis & tetanus) vaccine<br>4=Pentavalent vaccine (diphtheria, pertussis, tetanus, hepatitis B and haemophilus influenzae type B vaccines)<br>5=Hepatitis B vaccine<br>6=Hemophilus influenzae type B vaccine<br>7= MMR (measles, mumps, and rubella vaccine)<br>8=Yellow fever vaccine<br>9=Tetanus toxoid vaccine<br>10=HPV (human papillomavirus) vaccine<br>11=Hepatitis A vaccine<br>12=Hepatitis C vaccine<br>13=Typhoid fever vaccine<br>14=Rabies vaccine.<br>15=Pneumococcal conjugate vaccine<br>16=Cholera vaccine<br>17=Varicella vaccine<br>18=COVID-19 vaccine<br>19= Meningococcal vaccine<br>20=Others(specify)

Question	Response value/ coding or type
7.7.2. **Describe the reaction to vaccine**	1=Pain at injection site<br>2=Redness around injection site<br>3=swelling around injection site<br>4=bruise(haematoma) around injection site<br>5=Itching around injection site<br>6=warmth around injection site<br>7=Fever (hotness of body)<br>8=Chills (feeling of being cold)<br>9=Headache<br>10=nausea (feeling of wanting to vomit)<br>11=vomiting<br>12=Muscle ache<br>13=Joint pain<br>14=Malaise (feeling of weakness/not well)<br>15=fatigue (feeling tired)<br>16=urticaria (hives/weal/allergic rashes)<br>17= Anaphylactic shock<br>18=other (Specify)
8. COVID-19 vaccine exposure information	
8.1. Dose of **current** COVID-19 vaccine	1 = 1st vaccination<br>2 = 2nd vaccination<br>3 = 3rd vaccination<br>4 = 4th vaccination<br>5 = other (specify)
8.2. Date of **current** vaccination	dd/mm/yyyy
8.3. Time of **current** vaccination	HH:MM
8.4. Vaccine brand/ manufacturer	1= AstraZeneca<br>2= Pfizer<br>3= Johnson & Johnson<br>4= Moderna<br>5= Sputnik V<br>6= Sinopharm<br>7= Other (specify<br>8= Don't know
8.5. Vaccine batch number	Text
8.6. **Pre-populated based on response to 8.4**: Was a separate diluent required?	0 = No<br>1 = Yes
8.6.1. **If yes**: Diluent brand/ manufacturer	1= AstraZeneca<br>2= Pfizer<br>3= Johnson & Johnson<br>4= Moderna<br>5= Sputnik V<br>6= Sinopharm<br>7= Other (specify<br>8= Don't know

Question	Response value/ coding or type
8.6.2. Diluent batch number	Text
8.7. Co-administration of vaccine against any other disease other than COVID	0 = No<br>1 = Yes
8.7.1. **If yes**: Specify which disease was vaccinated against	Text
8.8. Received previous dose of COVID vaccine?	0 = No<br>1 = Yes
8.8.1. **If yes**: Number of previous doses	Text
8.8.2. Dose 1 confirmed using:	0 = Verbal report<br>1 = SMS<br>2 = Vaccination certificate
8.8.2.1. Dose 1 date	dd/mm/yyyy
8.8.2.2. Dose 1 vaccine brand/ manufacturer	1= AstraZeneca<br>2= Pfizer<br>3= Johnson & Johnson<br>4= Moderna<br>5= Sputnik V<br>6= Sinopharm<br>7= Other (specify<br>8= Don’t know
8.8.3. Dose 2 confirmed using:	0 = Verbal report<br>1 = SMS<br>2 = Vaccination certificate
8.8.3.1. Dose 2 date	dd/mm/yyyy
8.8.3.2. Dose 2 vacc/ine brand/ manufacturer	1= AstraZeneca<br>2= Pfizer<br>3= Johnson & Johnson<br>4= Moderna<br>5= Sputnik V<br>6= Sinopharm<br>7= Other (specify<br>8= Don’t know
8.8.4. Dose 3 confirmed using:	0 = Verbal report<br>1 = SMS<br>2 = Vaccination certificate
8.8.4.1. Dose 3 date	dd/mm/yyyy
8.8.4.2. Dose 3 vaccine brand/ manufacturer	1= AstraZeneca<br>2= Pfizer<br>3= Johnson & Johnson<br>4= Moderna<br>5= Sputnik V<br>6= Sinopharm<br>7= Other (specify<br>8= Don’t know

Pre-vaccine reactogenicity questionnaire, reactogenicity subset (if the response to Q5 is ‘yes’)

Question	Response value/ coding or type
9. **If Q5 is yes:** Pre-vaccine reactogenicity	
9.1. Unique ID for the record	Unique Numeric/ text/ alphanumeric
9.2. Date of record	dd/mm/yyyy
9.3. Did you feel **feverish** in the past 3 days?	0 = No<br>1 = Yes
9.3.1. **If yes:** Did you measure your temperature in the past 3 days?	0 = No<br>1 = Yes
9.3.2. **If yes:** What was your temperature?	1 = Below 38.0°C (below 100.4°F)<br>2 = 38.0°C to 38.4°C (100.4°F to 101.12 °F)<br>3 = 38.5°C to 38.9°C (101.3°F to 102.02°F)<br>4 = Higher than 39.0°C (higher than 102.2°F)<br>5= I can't remember
9.3.3. How did you measure the temperature?	1 = Oral (in the mouth)<br>2 = Rectum (in the anus)<br>3 = Armpit<br>4 = Ear<br>5 = Forehead
9.4. Did you feel **like vomiting (nausea)** in the past 3 days, or did you vomit?	0 = No<br>1 = Yes
9.4.1. **If yes:** How severe was the nausea/ vomiting?	1 = The nausea/vomiting did not interfere with my activities<br>2 = The nausea/vomiting somewhat interfered with my activities<br>3 = The nausea/vomiting was considerable and prevented my daily activities
9.5. Did you experience **a feeling or weakness or not feeling well (malaise)** in the past 3 days?	0 = No<br>1 = Yes
9.5.1. **If yes:** How severe was the malaise?	1 = The malaise did not interfere with my activities<br>2 = The malaise somewhat interfered with my activities<br>3 = The malaise was considerable and prevented my daily activities
9.6. Did you have **a feeling of being cold (chills)** in the past 3 days?	0 = No<br>1 = Yes
9.6.1. **If yes:** How severe were the chills?	1 = The chills did not interfere with my activities<br>2 = The chills somewhat interfered with my activities<br>3 = The chills were considerable and prevented my daily activities
9.7. Did you have a **headache** in the past 3 days?	0 = No<br>1 = Yes

Question	Response value/ coding or type
9.7.1. **If yes:** How bad was the headache?	1 = The headache did not interfere with my activities<br>2 = The headache somewhat interfered with my activities<br>3 = The headache was considerable and prevented my daily activities
9.8. Did you feel **joint pain** in the past 3 days?	0 = No<br>1 = Yes
9.8.1. **If yes:** How bad was the joint pain?	1 = The joint pain did not interfere with my activities<br>2 = The joint pain somewhat interfered with my activities<br>3 = The joint pain was considerable and prevented my daily activities
9.9. Did you have **muscle aches** in the past 3 days?	0 = No<br>1 = Yes
9.9.1. **If yes:** How bad were the muscle aches?	1 = The muscle aches did not interfere with my activities<br>2 = The muscle aches somewhat interfered with my activities<br>3 = The muscle aches were considerable and prevented my daily activities
9.10. Did you feel **tired (fatigued)** in the past 3 days?	0 = No<br>1 = Yes
9.10.1. **If yes:** How bad was the tiredness?	1 = The tiredness did not interfere with my activities<br>2 = The tiredness somewhat interfered with my activities<br>3 = The tiredness was considerable and prevented my daily activities

Cohort post-vaccine follow-up questionnaire (all participants)

**Questions to be automatically populated/ completed by study team**	**Response value/ coding or type**
10. Participant unique identifier (from baseline questionnaire)	Unique Numeric/ text/ alphanumeric
11. Identifier of the follow-up questionnaire	Unique Numeric/ text/ alphanumeric
12. Questionnaire completed?	0 = No<br>1 = Yes
12.1. **If no:** Reason why follow-up questionnaire was not completed	0 = No reason given<br>1 = The study takes too much time<br>2 = Not interested anymore<br>4 = Death<br>5 = Participant unreachable<br>6= Other (Specify)
12.1.1. **If death**: Reason for death	Text

*Questions that are shaded grey will not be included in the USSD-based questionnaire. Study staff will call participants answering 'yes' to hospitalization or COVID-19 diagnosis to collect the additional information in the grey-shaded sections.

**Questions to be completed by interviewing the participant via USSD-based or phone call-based questionnaire***	**Response value/ coding or type**
13. How many times did you seek medical care between date X and date Y? (e.g.at a local health centre or hospital)	0 = Did not seek medical care<br>1 = 1 times<br>2 = 2 times<br>3 = 3 times<br>4 = Other (Specify)
14. Were you hospitalized since **[**date of last contact**]?**	0 = No<br>1 = Yes
14.1. **If yes**: What was the date of hospital admission?	dd/mm/yyyy
14.2. Have you been discharged from hospital?	0 = No<br>1 = Yes
14.3. What was the date of hospital discharge?	dd/mm/yyyy
14.4. What was the reason for your hospitalization?	Text
14.5. What was the diagnosis by the healthcare provider?	Text
14.6. Picture/ attachment of discharge report available?	0 = No<br>1 = Yes
14.7. Name and place of hospital	Text
15. Were you diagnosed with COVID-19 by a healthcare professional?	0 = No<br>1 = Yes
15.1. **If yes**: Was the diagnosis based on a laboratory test?	0 = No<br>1 = Yes<br>2 = I do not know

15.2. What was the date of symptom onset?	dd/mm/yyyy
15.3. Was admission to the intensive care unit necessary?	0 = No<br>1 = Yes
16. Are you pregnant?	0 = No<br>1 = Yes<br>2 = Not applicable
17. Have you received another COVID-19 vaccine dose since last contact?	0 = No<br>1 = Yes
8.1. **If yes**: Dose of **current** COVID-19 vaccine?	1 = 2nd vaccination<br>2 = 3rd vaccination<br>3 = 4th vaccination<br>4= other (specify
8.2. Date of **current** vaccination	dd/mm/yyyy
8.3. Vaccine brand/manufacturer	1= AstraZeneca<br>2= Pfizer<br>3= Johnson & Johnson<br>4= Moderna<br>5= Sputnik V<br>6= Sinopharm<br>7= Other (specify<br>8= Don’t know

Reactogenicity: post-vaccine reactogenicity questionnaire (reactogenicity subset only)

*Questions that are shaded grey will not be included in the USSD-based questionnaire. Study staff will call participants answering ‘yes’ to any of the reactogenicity events to collect the additional information in the grey-shaded sections.

Question*	Response value/ coding or type
18. Participant unique ID (from baseline questionnaire)	Unique Numeric/ text/ alphanumeric
19. ID for the record of post-vaccination reactogenicity corresponding to each date	Unique Numeric/ text/ alphanumeric
20. Date of record	dd/mm/yyyy
21. Did you feel **feverish** yesterday?	0 = No<br>1 = Yes
21.1. **If yes:** Did you measure your temperature yesterday?	0 = No<br>1 = Yes
21.2. **If yes:** What was your temperature?	1 = Below 38.0°C (below 100.4°F)<br>2 = 38.0°C to 38.4°C (100.4°F to 101.12 °F)<br>3 = 38.5°C to 38.9°C (101.3°F to 102.02°F)<br>4 = Higher than 39.0°C (higher than 102.2°F)<br>5= I can’t remember
21.3. How did you measure the temperature?	1 = Oral (in the mouth)<br>2 = Rectum (in the anus)<br>3 = Armpit<br>4 = Ear<br>5 = Forehead
22. Did you feel **like vomiting (nausea)** yesterday, or did you vomit?	0 = No<br>1 = Yes
22.1. **If yes:** How severe was the nausea/ vomiting?	1 = The nausea/vomiting did not interfere with my activities<br>2 = The nausea/vomiting somewhat interfered with my activities<br>3 = The nausea/vomiting was considerable and prevented my daily activities
**23.** Did you experience **a feeling of weakness or not feeling well (malaise)** in the past 3 days?	0 = No<br>1 = Yes
23.1. **If yes:** How severe was the malaise?	1 = The malaise did not interfere with my activities<br>2 = The malaise somewhat interfered with my activities<br>3 = The malaise was considerable and prevented my daily activities

Question*	Response value/ coding or type
**24.** Did you have **a feeling of being cold (chills)** yesterday?	0 = No<br>1 = Yes
24.1. **If yes:** How severe were the chills?	1 = The chills did not interfere with my activities<br>2 = The chills somewhat interfered with my activities<br>3 = The chills were considerable and prevented my daily activities
**25.** Did you have a **headache** yesterday?	0 = No<br>1 = Yes
25.1. **If yes:** How bad was the headache?	1 = The headache did not interfere with my activities<br>2 = The headache somewhat interfered with my activities<br>3 = The headache was considerable and prevented my daily activities
**26.** Did you feel **joint pain** yesterday?	0 = No<br>1 = Yes
26.1. **If yes:** How bad was the joint pain?	1 = The joint pain did not interfere with my activities<br>2 = The joint pain somewhat interfered with my activities<br>3 = The joint pain was considerable and prevented my daily activities
27. Did you have **muscle aches** yesterday?	0 = No<br>1 = Yes
27.1. **If yes:** How bad were the muscle aches?	1 = The muscle aches did not interfere with my activities<br>2 = The muscle aches somewhat interfered with my activities<br>3 = The muscle aches were considerable and prevented my daily activities
28. Did you feel **tired (fatigued)** yesterday?	0 = No<br>1 = Yes
28.1. **If yes:** How bad was the tiredness?	1 = The tiredness did not interfere with my activities<br>2 = The tiredness somewhat interfered with my activities<br>3 = The tiredness was considerable and prevented my daily activities
29. Questionnaire completed?	0 = No<br>1 = Yes

Question*	Response value/ coding or type
**12.1 If no:** Reason why follow-up questionnaire was not completed	0 = No reason given<br>1 = The study takes too much time<br>2 = Not interested anymore<br>4 = Death<br>5 = Participant unreachable<br>6= Other (Specify)
29.1.1. **If death:** Reason for death	Text
