# Supplementary material for: Active vaccine safety surveillance: Experience from a prospective cohort event monitoring study of COVID-19 vaccines in Kenya
Source: PLOS Glob Public Health. 2025 Nov 17;5(11):e0005080. doi: 10.1371/journal.pgph.0005080 (PMC12622800; doi:10.1371/journal.pgph.0005080)
Supplement: Attachment — Submitted filename: Response to Reviewers.docx [file pgph.0005080.s023.docx]

Don Brian Odhiambo

KEMRI-Wellcome Trust Research Programme

Kilifi, Kenya

**Email:** [**dbrian@kemri-wellcome.org**](mailto:dbrian@kemri-wellcome.org)

**12 June 2025**

Executive Editor

PLOS Global Public Health

Dear Editor,

We are grateful to the editor and reviewers for their thorough and insightful feedback.

We have carefully considered and responded to each comment, as outlined below. We trust that our responses and the accompanying changes adequately address the concerns raised and contribute to a more robust presentation of our findings. Kindly let us know in case of any further comment or clarification.

a. **Response to comments from the editor:**

**Comment (i):** Figure 1: please (a) provide a direct link to the base layer of the map (i.e., the country or region border shape) and ensure this is also included in the figure legend; and (b) provide a link to the terms of use / license information for the base layer image or shapefile. We cannot publish proprietary or copyrighted maps (e.g. Google Maps, Mapquest) and the terms of use for your map base layer must be compatible with our CC-BY 4.0 license.
Note: if you created the map in a software program like R or ArcGIS, please locate and indicate the source of the basemap shapefile onto which data has been plotted.

**Response:**

**(a).** Direct link:

The base layer shapefiles used to plot Figure 1 were obtained from the Humanitarian Data Exchange at the following link: <https://data.humdata.org/dataset/cod-ab-ken>. The maps were created using ArcGIS Desktop 10.8.2, a GIS software developed by Esri, Redlands, CA, USA. This source has been cited in the legend of Figure 1.

**(b**). The shapefiles were originally provided by the Kenya National Bureau of Statistics (KNBS) and are distributed under the Creative Commons Attribution 4.0 International (CC BY 4.0) license. This license allows for unrestricted use, distribution, and reproduction in any medium, provided the original source is appropriately cited. The license details can be found at: <https://creativecommons.org/licenses/by/4.0/>.

**Comment (ii):** Please review your reference list to ensure that it is complete and correct. If you have cited papers that have been retracted, please include the rationale for doing so in the manuscript text or remove these references and replace them with relevant current references. Any changes to the reference list should be mentioned in the rebuttal letter that accompanies your revised manuscript. If you need to cite a retracted article, indicate the article’s retracted status in the References list and also include a citation and full reference for the retraction notice.

**Response:** We have reviewed the reference list and ensured that no retracted article has been cited. The reference list is complete and accurate. The reference list has been updated to reflect revisions to the manuscript in response to the comments raised though no new article has been added to the reference list.

b. **Response to Reviewer #1 comments:**

**Comment (i):** The types of vaccines for COVID-19 were not separately studied.

**Response:** We studied three COVID-19 vaccine brands (Pfizer, Johnson & Johnson and Moderna), which were the products administered during the study period in the participating vaccination centres. During statistical analyses, we evaluated the association between solicited systemic reactogenicity events and vaccine brand (Table 2). As described in the manuscript, the odds of reporting solicited systemic reactogenicity events were higher among individuals receiving Johnson & Johnson (aOR 2.05; 1.40-3.00) vaccine or Moderna (aOR 4.19; 2.34-7.51) vaccine than among Pfizer vaccine recipients. In addition, the associations between individual solicited reactogenicity events and vaccine brand are detailed in Supplementary Tables 8-15. The respective vaccine brand for each non-reactogenicity event is indicated in Supplementary Tables 16-18; for these events the sample size was insufficient to conduct vaccine brand-specific analyses.

**Comment (ii):** The exclusion criteria were not clearly outlined, as the symptoms that were supposed to be due to side effects of the vaccines were also common symptoms of many diseases.

**Response:** The eligibility criteria are described in the methods section (paragraph beginning on line 134). A standard set of solicited reactogenicity events was assessed, as per the standard WHO protocol employed for the study. At enrollment, participants were asked to retrospectively report any systemic symptoms they had experienced in the three days prior to enrolment (line 171). This was to distinguish systemic symptoms that existed prior to vaccination and those that occurred after vaccination (Supplementary Table 5). During statistical analyses, McNemar’s test was used to compare the frequency of post-vaccination solicited reactogenicity events to the baseline frequency (background) events to determine whether there were any statistically significant differences.

**Comment (iii):** The time of onset for the symptoms was not consistent for all participants.

**Response:** Solicited systemic reactogenicity events were tracked daily for seven days post-vaccination. Though some participants experienced these symptoms prior to enrolment (background events), most events occurred post-vaccination with events peaking within 1-2 days post-vaccination and resolving quickly (Supplementary Table 5). This is consistent with vaccine reactogenicity patterns from other COVID-19 vaccine reactogenicity studies.

**Comment (iv):** The authors' methods of analysing the data were not justifiable. For example, multivariate logistic regression analysis might not be a good statistical test for variables with two outcomes.

**Response:** Logistic regression is appropriate for a binary outcome such as the presence or absence of any solicited systemic reactogenicity event (composite outcome) or each solicited systemic event status (i.e. fever yes/no). We used multivariate logistic regression to adjust for potential confounders such as age, sex, comorbidities, vaccine dose, pregnancy status etc. while examining the association of the independent variable of interest with the respective outcome.

c. **Response to Reviewer #2 comments:**

**Comment (i):** Firstly, is it possible for the authors to outline the various levels of severity of the systemic events for the primary outcome as was used to determine their ability to interfere with the normal daily routines. I presume these must have had levels. If so, it is essential to outline the various levels of severity for the primary outcome as was determined by the authors.

**Response:** The severity levels were initially described within the legend of Figure 3 (Distribution of solicited systemic reactogenicity events by severity), the footnote of Supplementary Table 7, and in the study questionnaire included in the Supplement.

To make this more visible, we have added the definitions under the statistical analysis subheading of the methods section (Line 215).

**Comment (ii):** There is no clear reason or justification provided for the re-enrolment of the participant who was then followed up over two COVID-19 vaccine doses. Since they were not in the reactogenicity subset for either follow-up this makes their inclusion significantly different from the other participant. Even if their inclusion had negligible effects on overall findings, why was this done and what was the potential consequences on the overall results having in mind that one participant even thought treated independently is not sufficient for a conclusion and their inclusion raises issues of bias as they were characteristically different from the rest of the sample.

**Response:** The study protocol allowed re-enrolment when a previously enrolled individual returned for an additional COVID-19 dose during the study period to capture adverse events associated with each vaccine exposure. Only one person met this criterion during the study period, likely due to the relatively short timeframe of the study. The re-enrolled participant received two separate COVID-19 vaccine doses during the study period. Each dose was treated as an independent observation in the cohort analysis, as the safety profile of each dose was evaluated separately. This approach aligns with the WHO CEM protocol for COVID-19 vaccines, which permits follow-up of multiple doses per participant. Moreover, different COVID-19 doses are associated with different rates of post-vaccination events with many COVID-19 vaccine safety studies demonstrating that majority of events are observed after the administration of second vaccine dose.

Only 1000 participants were enrolled into the reactogenicity subset (daily 7-days follow up) before transitioning to the weekly follow-up of up to 13 weeks for hospitalizations and any other post-vaccination events. The remaining participants were enrolled into the non-reactogenicity subset which had weekly follow-up of up to 13 weeks. Therefore, this participant was not any different from the rest of the participants enrolled in the non-reactogenicity subset apart from having received two separate COVID-19 vaccine doses during the study period which was in line with standard CEM practice.

To enhance clarity, we have rewritten this statement in the Methods section under the study design and participants subheading to read “Former participants could be re-screened and re-enrolled if they received an additional COVID-19 vaccine dose *during the study period in line with the WHO protocol for CEM safety signal detection which allows follow-up of multiple doses per participant*.”

**Comment (iii):** Is it possible to add the implications of the limitations of the study to the overall findings. Is it possible to indicate to what extent the limitations affected the findings?

**Response:** It would be challenging to make definitive statements on the direction or magnitude of bias as little is known about the influence of the various limitations on mis-, under-, or over-reporting adverse events following adult vaccination in settings similar to where the study was conducted. We have added the following statement to reflect the overall impact of the study limitations to our findings: “Despite these limitations, our findings remained consistent with other COVID-19 vaccine safety studies as described earlier.” (line 398)
